# Autophagy in Silkworm Biology: Progress and Perspectives

**DOI:** 10.3390/biology15191688

**Published:** 2026-09-22

**Authors:** Lanlan Feng, Xin Zhou, Xia He

**Affiliations:** 1 Henan Sericulture Research Institute, Henan Academy of Agricultural of Sciences, Zhengzhou 450002, China; 2 College of Forestry, Henan Agricultural University, Zhengzhou 450002, China; zhouxin@stu.henau.edu.cn; 3 Henan Agricultural Product Quality Safety and Green Food Development Center, Zhengzhou 450002, China

**Keywords:** autophagy, silkworm, 20-hydroxyecdysone, metamorphosis, stress response, model organism

## Abstract

Autophagy is an evolutionarily conserved catabolic process essential for cellular quality control, metabolic adaptation, and developmental programming. The silkworm has emerged as a compelling model organism for investigating autophagy due to its well-characterized genome, ease of genetic manipulation, and significant homology with human disease-related genes. This review synthesizes current advances in silkworm autophagy research across four interconnected domains: molecular regulatory mechanisms, developmental functions, stress-induced responses, and disease-related implications. The steroid hormone 20-hydroxyecdysone (20E) orchestrates autophagy during metamorphosis through transcriptional upregulation of autophagy-related genes (*Atgs*) and mTOR inhibition. AMPK acts as a key energy sensor, phosphorylating BmAtg1c upon 20E stimulation or starvation. Functionally, autophagy participates in tissue remodeling, silk gland degeneration, and fat-body metabolism during development. Upon pathogenic infections (BmNPV, BmCPV, microsporidia) or xenobiotic stresses (pesticides, heavy metals), autophagy exhibits context-dependent duality—often promoting pathogen replication while also contributing to host defense. The interplay between autophagy and apoptosis serves as a critical molecular switch determining cellular fate under stress conditions. Collectively, these findings establish the silkworm as a versatile platform for decoding autophagy regulatory networks and their physiological significance.

## Simple Summary

Autophagy is a fundamental catabolic process governing cellular quality control, metabolic adaptation, and developmental programming across eukaryotes. The silkworm, domesticated for millennia as a silk-producing insect, holds substantial sericultural value. Concurrently, the silkworm has emerged as a well-established model organism, benefiting from a fully sequenced genome and considerable genetic homology to humans, thereby serving as a powerful platform for dissecting autophagic regulatory networks. This review summarizes current advances in silkworm autophagy research across four interconnected domains: (i) the functional characterization of autophagy-related genes; (ii) the roles of autophagy in silkworm metamorphosis and development; (iii) autophagic responses to various environmental stressors; and (iv) the potential of the silkworm as a model organism for elucidating autophagy–apoptosis crosstalk in disease contexts. Collectively, this review provides a foundational basis for future advancements in sericulture, disease management, and drug development.

## 1. Introduction

More than six decades have passed since Christian de Duve coined the term ‘autophagy’. Nevertheless, owing to the fundamental importance of this process in living organisms, research on autophagy in both animals and plants continues to increase year by year [1]. Autophagy is an evolutionarily conserved catabolic process that mediates the delivery of cytoplasmic constituents, including damaged organelles and protein aggregates, to the lysosome for degradation [2]. Rather than functioning solely as a mechanism for wholesale cellular clearance, autophagy operates as a dynamic recycling system that regenerates metabolic precursors and energy, thereby supporting cellular renovation, adaptation, and homeostasis [3]. This process is essential for intracellular quality control, metabolic adaptation, and proper execution of developmental and differentiation programs [4,5]. While autophagy generally serves a cytoprotective role, dysregulation of autophagic machinery or excessive autophagic flux can contribute to cell death [6]. Consequently, perturbation of autophagic function, whether through impairment or hyperactivation, has been implicated in the pathogenesis of various human diseases, including neurodegenerative disorders such as Parkinson’s disease and inflammatory conditions such as Crohn’s disease [7]. Yoshinori Ohsumi was awarded the 2016 Nobel Prize in Physiology or Medicine for elucidating the core molecular mechanisms of autophagy through pioneering genetic screening [8,9]. This year marks the 10th anniversary of his receipt of the Nobel Prize.

Autophagy is categorized into three primary types: macroautophagy, microautophagy, and chaperone-mediated autophagy (CMA). Macroautophagy is characterized by membrane elongation to sequester cytoplasmic components, while microautophagy involves direct invagination of the lysosomal membrane. Both of these pathways can mediate either selective or non-selective degradation. In contrast, CMA selectively targets cytosolic proteins for lysosomal degradation without requiring membrane deformation [10,11]. For macroautophagy, existing studies have shown that the biogenesis of autophagosomes depends on five core Atg complexes, which are evolutionarily conserved across plants and animals: the Atg1/Ulk1 kinase complex, the Atg9 complex, the Atg6/Beclin1-VPS15-VPS34 class III PI3K complex, the Atg5-Atg12 conjugate, and the Atg8/LC3 conjugation system. Among these, only Atg8 (LC3 in animals) becomes covalently conjugated to phosphatidylethanolamine (PE) on the autophagosomal membrane, a modification that renders it a widely utilized marker for detecting autophagosome structures and monitoring autophagic flux [12,13]. In yeast, more than 30 Atg proteins have been identified to participate in macroautophagy, and the majority of these are evolutionarily conserved in plants [14]. In *Drosophila melanogaster*, approximately 20 Atg proteins have been identified [15]. In addition, autophagy exhibits a paradoxical, context-dependent duality in cancer. On one hand, it acts as a cytoprotective mechanism, promoting cancer cell survival and therapeutic resistance by suppressing the effects of anti-cancer agents. On the other hand, the heightened autophagy induced by certain anti-cancer drugs can trigger autophagy-mediated cell death. Consequently, while some advanced cancers become dependent on autophagy for survival, autophagy manipulation can either promote or suppress tumor progression depending on cancer type, origin, and treatment context [16,17].

For over five millennia, silkworms (*Bombyx mori* L.) have maintained a close relationship with humans, contributing substantially to human welfare [18]. In detail, silkworms not only produce silk but also serve as a medicinal resource and feed ingredient, conferring health benefits to both humans and animals [19,20,21]. In addition, the silkworm offers multiple practical advantages as a model organism in life sciences, including cost-effective rearing, high fecundity, a brief life cycle, and a well-characterized genome. Its rich genetic resources and substantial sequence homology with human hereditary disease-related genes further support its utility as an alternative platform for investigating human pathologies [22]. Beyond disease modeling, the silkworm has demonstrated value in antimicrobial agent screening, environmental safety assessment, and antitumor research, highlighting its broad applicability and potential in biomedical and toxicological studies [23,24,25]. Furthermore, silkworms have been repurposed in modern biotechnology as a cost-effective and scalable biofactory for recombinant-protein expression [18]. Another study has demonstrated that Relish directly regulates *ApAtg5* transcription to mediate anti-microbial autophagy, revealing that autophagy is transcriptionally controlled by the innate immune pathway to eliminate Gram-negative bacteria, thereby contributing to immunologic homeostasis in *Antheraea pernyi* [26].

Research on autophagy in the silkworm has steadily increased over recent years. However, the findings to date remain somewhat fragmented. In this review, we synthesize the current knowledge by focusing on four key areas: the molecular mechanisms of autophagy, its role in silkworm development, its involvement in stress responses, and its regulation in the context of disease. Our goal is to provide a cohesive overview of the progress in silkworm autophagy research. Furthermore, we explore the potential of modulating autophagy to enhance silk production, develop antiviral agents, assess the toxicity of environmental pollutants, and screen for anti-aging pharmaceuticals.

## 2. Molecular Mechanism of Autophagy in the Silkworm

In the silkworm, 20-hydroxyecdysone (20E) signaling enhances autophagy through multiple pathways, including direct upregulation of autophagy-related and V-ATPase genes, inhibition of the mTOR pathway, and deacetylation of Atg proteins mediated by dephosphorylation of the histone deacetylase BmRpd3 [27,28,29,30]. Post-translational modifications are chemical changes to proteins after biosynthesis that alter their properties. Current studies in the silkworm have shown that acetylation of BmAtg4, BmAtg7, and BmAtg8 suppresses autophagy, whereas phosphorylation of BmAtg1, Tip60, and Rpd3/HDAC1 enhances autophagy [31,32,33]. BmAtg1 exhibits tissue- and stage-specific expression patterns in silkworm, with comparable transcript levels in the midgut, fat body, and Malpighian tubules, higher expression in the gonads, heterogeneous distribution across the anterior, middle, and posterior silk glands at the end of the larval stage, and starvation-induced upregulation in the fat body but not in the midgut [34]. AMPK activates BmAtg1c by phosphorylating its evolutionarily conserved Ser269 and Ser270 residues upon 20E stimulation or starvation, and this phosphorylation is indispensable for autophagy induction during larval–pupal metamorphosis [35]. Remarkably, activation of the starvation–AMPK axis promotes BmTip60 expression, a requisite step for autophagy induction in silkworms, offering new perspectives on the autophagic regulatory network in this species [36]. BmAtg1 and BmAtg8 play critical roles in mediating autophagic degradation of intracellular lipids. RNAi-mediated silencing of these genes inhibits autophagy, whereas their overexpression promotes lipid degradation [37]. 

BmAtg3 serves as a direct molecular partner of the phagocytic receptor Draper. The Draper–BmAtg3 interaction positively regulates autophagic activity, thereby promoting the degradation of the middle silk gland during metamorphosis [38]. Inhibition of vacuolar-type ATPase (V-ATPase) by bafilomycin A1 suppressed the expression of autophagy-related genes *BmAtg5*, *BmAtg6*, and *BmAtg8* in BmE cells, indicating that V-ATPase positively regulates autophagy in silkworm cells [39]. *BmAtg7* is a core autophagy-related gene that is highly expressed in metabolic and developmental tissues, including the posterior silk gland, fat body, and epidermis. Depletion of *BmAtg7* downregulates *BmAtg8* and *BmEcr* expression while upregulating *BmJHBP2*, and delays the larval–pupal transition without completely blocking metamorphosis [40]. The crystal structure of silkworm BmAtg8 was resolved at 2.40 Å resolution in 2010 [41]. In Bme cells, exposure to high pH potently induces the lipidation of BmAtg8 (conversion to BmAtg8-PE), an effect that is even stronger than that observed under amino acid starvation [42]. BmAtg8 and BmAtg12 are core components of the ubiquitin-like conjugation systems essential for autophagosome formation in the silkworm. Their time-course transcriptional upregulation in the silk gland from day 1 to day 8 of the fifth instar indicates that autophagy is progressively activated during silk gland maturation [43]. Moreover, mutations of acetylation sites within the BmAtg8-PE ubiquitin-like system components (BmAtg3, BmAtg4, and BmAtg8) cause their redistribution from the nucleus to the cytoplasm, which is accompanied by enhanced autophagic activity [44]. Mechanistically, acetylation of BmAtg8 at residue K13 weakens the interaction between BmAtg8 and BmAtg7, which in turn impairs the conjugation of BmAtg8 to PE and ultimately attenuates cell rescue during stress [45].

BmAtg13 is essential for autophagy induced by 20E or starvation, as its gene expression is upregulated by 20E treatment, and its protein level undergoes changes along with nucleocytoplasmic translocation during larval–pupal metamorphosis [46]. Autophagy degrades p62 bodies that are formed through the self-association of the adaptor protein Bmp62 with ubiquitinated proteins [47]. Overexpression of BmAL1 promotes lysosomal acidification and enhances autophagy, whereas BmAL1 knockout or knockdown inhibits lysosomal acidification and autophagic flux [48]. The list of the principal autophagy-related genes and their regulation in silkworm is summarized in Table 1 and Figure 1.

## 3. Autophagy in Silkworm Development

The establishment of morphological, biochemical, and molecular methods to monitor autophagy in silkworm organs provides a foundation for further studies on the physiological functions of this process in silkworm [49,50]. In the silkworm midgut during metamorphosis, autophagy is induced by the 20E commitment peak at the end of the last larval stage and plays a pro-survival role, while apoptosis, which onsets later with the 20E metamorphic peak, drives epithelial degradation. These findings demonstrate that autophagy precedes apoptosis and serves a protective role, rather than mediating autophagic cell death [51]. Autophagy is essential for tissue remodeling during silkworm development, and its activity is modulated by crosstalk between BmAtg proteins and caspases. BmCasp8L acts as a positive regulator of autophagy by inhibiting BmDREDD-mediated cleavage of BmAtg6, thereby facilitating autophagic flux [52]. In addition, autophagy plays an essential role in the formation of the pupal midgut in silkworms, as its pharmacological inhibition leads to morphological abnormalities during pupal epithelium development. This process involves the regulation of autophagy-related genes (*BmAtg8* and *BmAtg12)* and lysosomal enzyme activity within midgut stem cells [53]. In another study, during the larval–adult transformation of silkworms, autophagy is activated from the wandering stage and reaches high activity during the spinning and prepupal stages, as demonstrated by specific autophagic markers. This process recycles molecules from degenerating larval midgut cells and supplies nutrients to the animal during the non-feeding period [54]. Furthermore, during pupal–adult metamorphosis, autophagic cell death contributes to the disintegration of the PV fat body, as evidenced by the presence of giant autophagic vacuoles, cytoplasmic budding, and reduced mitochondrial numbers observed via electron microscopy [55]. During silkworm metamorphosis, autophagy in the midgut precedes apoptosis, with the cleavage of BmAtg5 and BmAtg6 into BmAtg5-tN and BmAtg6-C serving as a key mechanism that mediates apoptosis following the autophagic response [56,57]. The microRNA miR-34-5p participates in 20E-induced autophagy in silkworms through regulation of its target gene *BmAtg1.* These findings offer insights into the complex interplay among miRNAs, 20E signaling, and autophagic processes during fat-body remodeling and insect metamorphosis [58].

Moreover, autophagy participates in the degeneration of the silkworm silk gland, and autophagy persists for several days, progressively degrading the cells of the silk gland, while apoptosis is triggered exclusively during the terminal phase of silk gland degradation [59,60,61,62,63,64]. In detail, during silkworm metamorphosis from mature silkworm to pupal day 1, autophagy is significantly increased in the posterior silk gland from the MS to PP1 periods but decreases thereafter, occurring prior to apoptosis and playing a role in the programmed cell death process [65]. In particular, autophagy-mediated cell death involving BmAtg3, BmAtg5, and BmAtg12 is essential for the proper progression of posterior silk gland degeneration, as their knockdown reduces autophagy, alters apoptosis, and impairs lysosomal function [66]. During the transition from molt to intermolt in silkworms, autophagy-related pathways are activated, as evidenced by increased autophagosomes and lysosomes in silk gland cells, and contribute to silk gland renewal [67]. Transcriptional analysis reveals that *POUM2* homeostasis regulates the expression of autophagy-related genes, indicating that autophagy is involved in anterior silk gland intimal remodeling and cell fate determination during development and metamorphosis in the silkworm [68]. Knockdown of *BmADK* downregulates *BmAtg8* expression, indicating that *BmADK* positively regulates autophagy in the silkworm silk gland during metamorphosis [69].

Knockout of *BmGDAP2* significantly prolongs the growth cycle of silkworms by affecting genes involved in autophagy, apoptosis, and hormone regulation. These findings suggest that *BmGDAP2* plays a critical role in modulating developmental progression, potentially through autophagy-related pathways [70]. In homozygous mutants of the *fibroin light chain* (*Fib-L*) gene, autophagy, apoptosis, and the heat shock response were concurrently activated in the posterior silk glands, leading to premature degradation of the posterior silk glands in silkworms [71]. In parallel, the truncated fibroin heavy chain (FibH) in naked pupa (Nd) mutants disrupts cellular homeostasis in the posterior silk gland, leading to significant upregulation of autophagy-related genes, as evidenced by transcriptomic analysis [72]. Additionally, autophagy is activated early upon accumulation of abnormal Fib-H protein and Fib-H-like protein, promoting the rapid degradation of both abnormal proteins and silk gland cells [73,74,75]. During silkworm vitellogenesis, autophagy operates in synergy with apoptosis to facilitate the efficient and rapid clearance of the degenerated nurse cell cluster, as evidenced by the formation of autolysosomes in stage 7 nurse cells prior to the appearance of late apoptotic markers [76]. Notably, the autophagy pathway was identified as one of the significantly enriched biological pathways in diapause eggs of silkworms based on transcriptome analysis, suggesting its potential involvement in the regulation of embryonic diapause [77]. Autophagy-related factors, together with exosomes and under the regulation of 20E, mediate the stage-specific transport of mulberry-derived urease from the midgut to the hemolymph in silkworm larvae [78]. In conclusion, the roles of autophagy in regulating the metamorphic development of the silkworm are summarized in Figure 2.

## 4. Autophagy Response in Silkworm Under Stress Conditions

A broad range of stimuli, including viral pathogens, chemical pollutants, microbial parasites, and physicochemical stresses, can trigger autophagy in silkworm cells (Figure 3).

### 4.1. Biotic Stresses

*Bombyx mori* Nuclear Polyhedrosis Virus (BmNPV) infection induces host cell autophagy, accompanied by mitochondrial damage and mitophagy, and this virus-induced autophagic response promotes viral replication in silkworm cells [79,80]. Particularly, BmAtg5 positively regulates BmNPV replication through the promotion of autophagic flux, as evidenced by increased VP39 expression and viral DNA accumulation upon BmAtg5 upregulation and reduced viral replication upon its silencing [81]. BmAtg7 and BmAtg9 positively regulate BmNPV infection by contributing to the autophagy level induced by viral infection, thereby promoting viral gene expression [82]. Bmced6 promotes BmNPV infection by enhancing virus-induced autophagy, as evidenced by increased autophagosome formation and BmAtg8 lipidation, while simultaneously disrupting mitochondrial homeostasis, leading to decreased mitochondrial membrane potential and dysregulated ROS production [83]. The *BmAtg13* overexpression transgenic line facilitates BmNPV replication and elevates the mortality rate of infected silkworms, in contrast to the *BmAtg13* knockout transgenic line, which exhibits reduced viral replication [84]. Additionally, BmNPV enhances exogenous amino acid uptake by inducing the expression of the arginine transporter Slc7a6 and activating mitochondrial autophagy to supplement intracellular amino acids, thereby maintaining intracellular amino acid levels and facilitating viral proliferation [85]. However, BmAtg3, which is upregulated upon BmNPV infection, exerts a protective effect against oxidative damage in silkworm cells. This finding highlights a critical role of BmAtg3 in counteracting virus-induced oxidative stress as part of the innate immune response [86]. Overexpression of *BmFoxO* enhances antiviral resistance against BmNPV in silkworms by upregulating the expression of both BmPEPCK-2 and the autophagy-related genes *BmAtg6*, *BmAtg7,* and *BmAtg8* [87]. In addition, both *BmAtg4a* and *BmAtg4b* peak during larval–pupal metamorphosis when autophagy occurs robustly, and their overexpression slightly promotes basal autophagy but inhibits BmNPV-induced autophagy and subsequent viral proliferation [88]. Another study demonstrated that overexpression of BmPAT4 activates BmTORC1 to suppress autophagy in silkworm cells, thereby restricting BmNPV infection and replication, whereas BmPAT4 downregulation enhances autophagy and promotes viral proliferation [89]. Infection with BmNPV also induces time-dependent accumulation of reactive oxygen species (ROS), which in turn upregulates the expression of the ferritin heavy-chain homolog (BmFerHCH) and modulates autophagy in BmN cells [90]. 

Using iTRAQ-based proteomic analysis, a putative p62/sequestosome-1 protein (BGIBMGA002620-PA) was identified to be upregulated at both the mRNA and protein levels following *Bombyx mori* cytoplasmic nucleopolyhedrosis virus (BmCPV) infection, suggesting that autophagy, and particularly apoptosis, may play a critical role in the midgut defense response against viral invasion [91]. Autophagy exhibited only a weak response to BmCPV infection and was insufficient to block viral invasion or replication [92]. BmCPV infection induces autophagosome formation and mitochondrial structural damage in the silkworm midgut, and the subsequent mitophagy promotes viral proliferation [93]. Moreover, the BmCPV-derived circRNA-encoded peptide vsp21 suppresses viral replication by interacting with ubiquitin carboxyl-terminal hydrolase (UCH), which enhances the ubiquitination and degradation of phospho-IκB-α, thereby activating the NF-κB/autophagy pathway [94]. BmCPV infection also induces differential expression of host lncRNAs, which may regulate the replication of the virus by targeting key genes involved in autophagy and antiviral responses, including *BmAtg3* [95]. *Bombyx mori* bidensovirus (BmBDV) infects the silkworm midgut and causes chronic flacherie disease. RNA-seq analysis showed that several genes related to autophagy are induced in the silkworm midgut, suggesting their potential involvement in the antiviral immune response [96].

Upon *Nosema bombycis* infection, the expression of BmSTING and the autophagy marker protein BmAtg8 is concurrently upregulated in silkworm BmE cell lines [97]. Meanwhile, differentially expressed mRNAs (DEmRNAs) in silkworm midguts are predominantly involved in key biological pathways, including metabolism, apoptosis, and autophagy, following *Nosema bombycis* infection [98]. In addition, the expression of *BmATG8*, a key regulator of autophagy, was induced in the ovaries of silkworm following *Nosema bombycis* infection, suggesting that the parasite may trigger autophagic processes in the host [99]. In response to parasitism by *Exorista bombycis*, the expression of *BmAtg5L* in the silkworm integumental epithelium was induced in an age-dependent manner, with significant early upregulation associated with the onset of autophagy [100].

### 4.2. Abiotic Stresses

The interplay between autophagy and apoptosis critically determines cellular fate in silkworm under pesticide stress, with their crosstalk acting as a key molecular switch that integrates stress signals and dictates whether cells survive or die [101]. Regarding the impacts caused by pesticide overuse, low-concentration chlorantraniliprole exposure results in significant chlorantraniliprole residues in the silk gland of *Bombyx mori*, accompanied by decreased Ca^2+^-ATPase activity, activation of AMPK signaling, and upregulation of downstream autophagy-related genes (e.g., *BmAtg1*, *BmAtg5*, *BmAtg8*), with *BmAtg8* expression further confirming the induction of autophagy [102]. Other studies showed that exposure of silkworms to sublethal chlorantraniliprole induces intracellular Ca^2+^ release, which inhibits the oxidative phosphorylation pathway and causes mitochondrial dysfunction, thereby significantly upregulating the transcript levels of autophagy-related genes *BmAtg6* and *BmAtg8* as well as the protein levels of BmAtg8-PE and BmAtg7 in the midgut and silk gland of *Bombyx mori* [103,104]. Pyriproxyfen treatment disrupted hormonal regulation in silkworms by activating the juvenile hormone signaling pathway and inhibiting the 20E signaling pathway. This disruption subsequently upregulated autophagy-related genes including *BmAtg5*, *BmAtg6*, *BmAtg12*, and *BmAtg16*, and also activated autophagy-related signaling pathways such as the NOD-like receptor, ultimately promoting autophagy in the posterior silk gland [105]. Furthermore, ultrastructural analysis revealed that exposure to dinotefuran induces an increase in autophagic vesicles within the silk gland of the silkworm [106]. Another study revealed that dinotefuran exposure induces oxidative stress within the silkworm midgut and fat body, as evidenced by the upregulation of the autophagic markers BmAtg8-PE and BmAtg6, and a concurrent reduction in sequestosome 1 (p62) levels, collectively signifying the activation of autophagic flux. Importantly, pharmacological inhibition of autophagy significantly attenuates the expression of both BmAtg6 and the pro-apoptotic protein BmDredd. These findings support a model in which dinotefuran-induced autophagy promotes apoptosis in silkworm tissues [107]. Moreover, short-term exposure to a low concentration of indoxacarb induces autophagy in the midgut cells of silkworms, as evidenced by the significant upregulation of *BmAtg2*, *BmAtg8*, and *BmAtg9* transcription, increased BmAtg8 and BmAtg8-PE protein levels, and positive MDC staining [108]. In response to methyl-thiophanate treatment, silkworms activate autophagy in the midgut, a process linked to oxidative stress and changes in metabolism, indicating its role in mediating the cellular response to this fungicide [109]. 

Starvation induces Ca^2+^ overload in silkworm midgut cells by upregulating Ca^2+^ release channel-associated genes and suppressing Ca^2+^ efflux genes, which activates autophagy as an early response. Upon prolonged starvation, increased NtAtg5 protein levels facilitate the transition from autophagy to apoptosis, revealing that Ca^2+^ overload also promotes calpain-mediated apoptosis [110,111]. In the thermotolerant silkworm strain Liangguang NO.2, high-temperature stress upregulated *BmAtg5* expression as well as 20E titers and ultraspiracle 1 expression, suggesting that ecdysone signaling may contribute to the induction of autophagy. Conversely, the thermosensitive strain Jingsong × Haoyue exhibited reduced *BmAtg12* expression under heat stress, indicating that strain-specific autophagic responses are associated with differential thermotolerance in silkworms [112]. High-temperature treatment also triggers Ca^2+^-mediated autophagy, which is further shifted toward apoptosis, as evidenced by elevated levels of BmAtg5, caspase-3, and cleaved caspase-3 [113]. In addition, the imbalanced amino acid profile of formula feed leads to reduced DNA proliferation, decreased transcript levels of Fib-H, Fib-L, and P25, and mild autophagic activity in the posterior silk gland, collectively resulting in lower cocoon shell weight and ratio in silkworms [114]. Chronic copper exposure activates the PI3K/AKT/mTOR signaling pathway, which triggers abnormal autophagy in silkworms [115]. Autophagy, together with the Toll and IMD pathways, plays a pollutant-specific role in mediating the toxic effects of fluoride and cadmium in silkworms, as revealed by CRISPR-based functional genomic screening [116].

## 5. Autophagy and Disease: Insights from the Silkworm Model

Autophagy research in Drosophila melanogaster has advanced significantly, revealing its complex and context-dependent roles in development, the stress response, and neurodegeneration. In Drosophila melanogaster, autophagy is dynamically regulated during metamorphosis, mediating the programmed degradation of larval tissues such as the midgut and salivary glands [117,118]. Hormonal signals, including ecdysone and juvenile hormone, coordinate autophagy through pathways involving JNK signaling [118]. Beyond development, autophagy in the Drosophila melanogaster brain plays a critical role in synaptic remodeling and organismal resilience. Local impairment of autophagy in the mushroom body triggers brain-wide presynaptic active zone remodeling, characterized by altered scaffold protein and ion channel composition, which promotes sleep and extends lifespan [119]. In the ovary, autophagy exhibits remarkable cell-type specificity: it is dispensable in germline stem cells but essential in niche cells for their maintenance during aging [120]. Genetically tractable disease models, such as dwdr45 knockout flies, have further established Drosophila melanogaster as a powerful platform for dissecting autophagy’s role in neurodegeneration [121].

While *Drosophila melanogaster* offers good genetic tools, the silkworm provides unique advantages as a complementary model. The silkworm’s large size facilitates tissue-specific biochemical and histological analyses, particularly of the silk gland—a specialized organ that undergoes massive autophagic degradation during metamorphosis. Moreover, the silkworm is a valuable model for investigating intestinal stem cells (ISCs) as well as cell death-related processes including apoptosis and autophagy [122]. The silkworm also serves as an effective in vivo model for evaluating nanotoxicity in gonads, as demonstrated by the induction of germ cell death and malformations via mitochondrial and lysosomal pathways following exposure to cadmium telluride quantum dots (CdTe-QDs) [123]. The silkworm represents a valuable animal model for hyperproteinemia, featuring a controllable plasma protein concentration without the confounding effects of primary diseases due to its open circulatory system where organs are directly bathed in hemolymph. In addition, autophagy induced by *BmPer* knockdown is associated with a slowdown of the cell cycle and reduced cell proliferation, suggesting a regulatory link between the autophagic process and cell cycle progression [124]. Pathogenesis studies reveal that hyperproteinemia attenuates an endocrine hormone, thereby suppressing cell autophagy and apoptosis, which consequently prevents fat-body remodeling during metamorphosis [125]. For example, in the silkworm model, SiNPs induce autophagy and apoptosis specifically in differentiated hemocytes via the lysosomal/mitochondrial pathway, but do not severely affect hematopoietic stem cells (prohemocytes) or the hematopoietic organ, resulting in self-limiting damage to hematopoiesis [126]. In addition, the silkworm larva serves as a sensitive and effective in vivo model for assessing the biotoxicity of ionic liquids, as exposure induces significant physiological, biochemical, and transcriptional alterations in the midgut and silk glands [127].

In the silkworm *Nd mutant*, autophagic flux is impaired due to defective lysosomal acidification, as evidenced by the accumulation of SQSTM1/p62 and ubiquitinated proteins despite upregulated BmAtg8-PE. This dysfunction prevents efficient degradation of autolysosomal substrates, leading to protein aggregation and posterior silk gland degeneration [128]. Autophagy serves as an accessory immune effector mechanism in insects, contributing to the host’s defense against pathogen invasion during insect–pathogen interactions [129]. Cathepsin L-like protease regulates both innate immunity and anti-microbial autophagy in silkworms, as suppression of this protease alters the expression of immune-related genes in the Toll and IMD pathways as well as autophagy-related genes including *BmAtg6*, *BmAtg8*, *VAMP2*, *Vps4*, and *syntaxin* [130]. 

Silkworm NPC2 protein inhibits the growth of FM3A murine breast cancer cells by inducing autophagy, as evidenced by increased BmAtg8-PE levels and BmAtg8-dot formation. This autophagic induction involves AMPK phosphorylation alongside decreased Akt and mTOR phosphorylation, and the anti-proliferative effect is attenuated by the autophagy inhibitor chloroquine [131]. Silkworm storage protein 1 (SP1) suppresses autophagy-mediated apoptosis by downregulating basal Beclin-1 expression, thereby inhibiting upstream events in the autophagy pathway, as well as by reducing BmAtg8-to-BmAtg8-PE conversion and BmAtg7 expression. Additionally, SP1 attenuates caspase-3 activation and reactive oxygen species generation under starvation conditions [132].

## 6. Perspectives

Despite substantial progress, several fundamental questions in silkworm autophagy research remain unresolved. First, the precise molecular choreography governing the transition from autophagy to apoptosis during metamorphosis requires deeper elucidation, particularly regarding caspase-mediated BmAtg cleavage and the spatiotemporal dynamics of autophagic flux in vivo. Second, while numerous *BmAtg* genes have been identified, their tissue-specific interactomes and context-dependent regulatory partners remain largely unexplored. Integrating CRISPR-based functional screening with proteomic approaches will likely accelerate the discovery of novel autophagy modulators. Third, the paradoxical role of virus-induced autophagy, simultaneously promoting viral replication while contributing to host survival, warrants further mechanistic dissection to develop antiviral strategies that selectively attenuate pathogenic autophagy without compromising essential cellular functions. Fourth, the application of silkworm autophagy research to translational fields holds considerable promise. Modulating autophagic activity may enhance silk protein production in sericulture, improve the efficacy of recombinant-protein expression systems, and enable high-throughput screening of environmental toxicants and anti-aging compounds. Finally, given the substantial sequence and pathway conservation between silkworms and mammals, insights from this model may inform the understanding of autophagy-related human diseases, including neurodegenerative disorders and cancer. Future studies integrating multi-omics analyses, advanced imaging techniques, and genetic tools will be instrumental in unraveling the complexity of autophagic regulation and its pleiotropic roles in development, stress adaptation, and disease.

## 7. Conclusions

In conclusion, this review consolidates current knowledge on autophagy in the silkworm, across molecular, developmental, stress-responsive, and disease-related contexts. At the molecular level, 20E and nutrient sensing pathways, particularly AMPK–BmAtg1c signaling, converge with post-translational modifications to orchestrate autophagic activity, while core BmAtg proteins execute autophagosome formation and cargo degradation. Functionally, autophagy precedes apoptosis and is indispensable for tissue remodeling, silk gland degeneration, fat-body metabolism, and midgut reconstruction during metamorphosis. Under biotic and abiotic stresses, autophagy displays context-dependent duality, either promoting pathogen replication or contributing to host defense, and its crosstalk with apoptosis constitutes a critical switch determining cell fate. The silkworm further offers a tractable in vivo platform for dissecting autophagy–apoptosis interplay in disease and toxicological settings. Collectively, these findings establish a coherent framework for silkworm autophagy research and highlight its implications for sericulture improvement, antiviral and antimicrobial strategies, environmental safety assessment, and translational studies of autophagy-related human diseases.

## Figures and Tables

**Figure 1:**
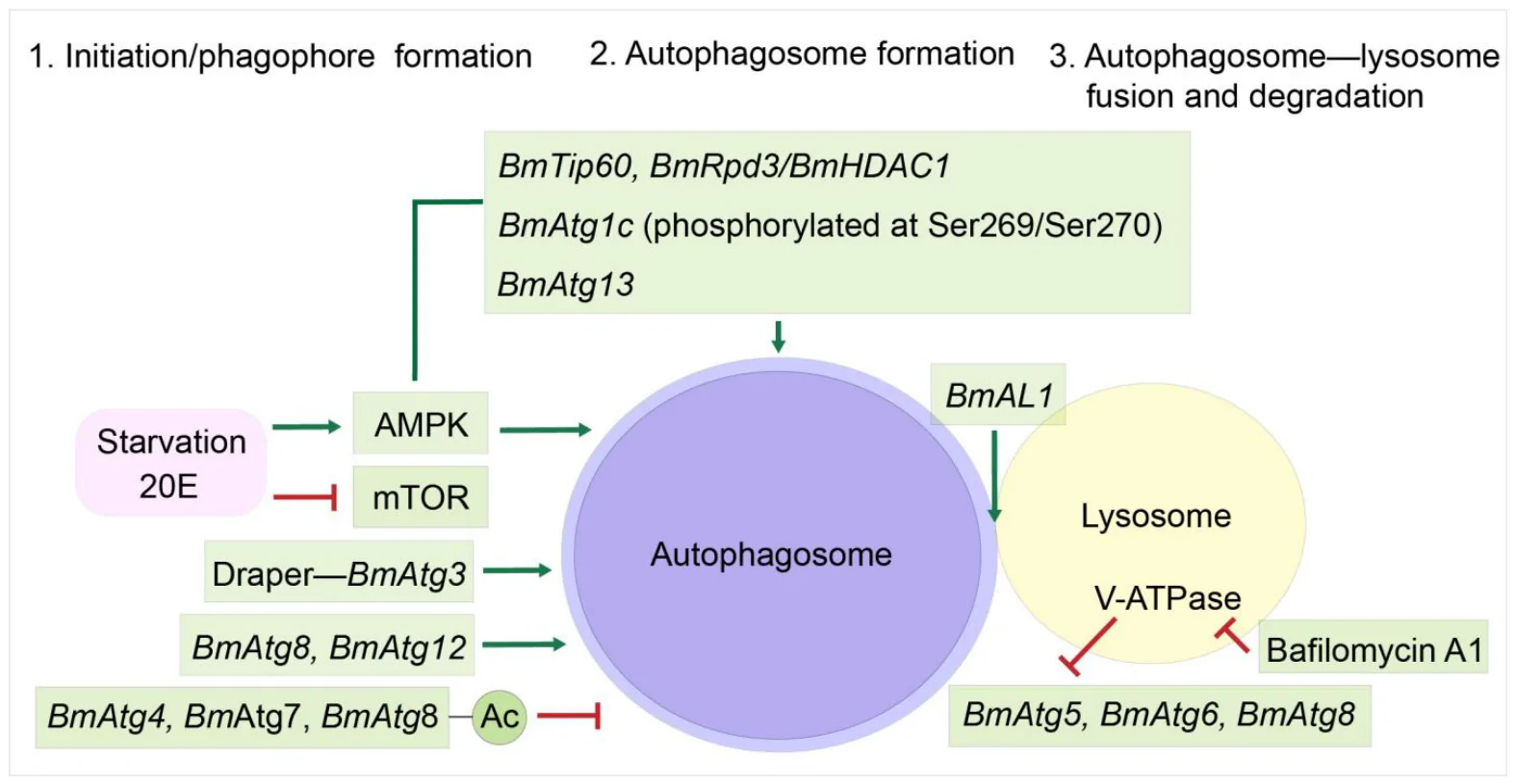
Functional illustration of autophagy-related genes in the silkworm. Autophagy is governed by hormonal signals, nutrient sensors, and post-translational modifications (PTMs). 20E induces autophagy during larval–pupal metamorphosis by upregulating BmAtgs and V-ATPase, inhibiting mTOR, and promoting BmAtg deacetylation via BmRpd3. AMPK phosphorylates BmAtg1c upon 20E or starvation, and upregulates BmTip60. PTMs exert bidirectional control: acetylation of BmAtg4, 7 and 8 suppresses autophagy, whereas phosphorylation of BmAtg1, BmTip60, and BmRpd3 enhances it; BmAtg8 acetylation impairs BmAtg8-PE conjugation. Core BmAtgs show distinct functions: BmAtg1 mediates fat-body starvation responses; Draper-BmAtg3 promotes middle silk gland degradation; BmAtg7 is required for larval–pupal transition; BmAtg13 is essential for 20E- and starvation-induced autophagy; BmAtg8 and 12 drive autophagosome formation. V-ATPase positively regulates autophagy, and BmAL1 promotes lysosomal acidification and flux. Green and red lines represent the promotional and inhibitory effects, respectively; Pink indicates stimulation; green indicates different genes; purple indicates autophagosomes; yellow indicates lysosomes.

**Figure 2:**
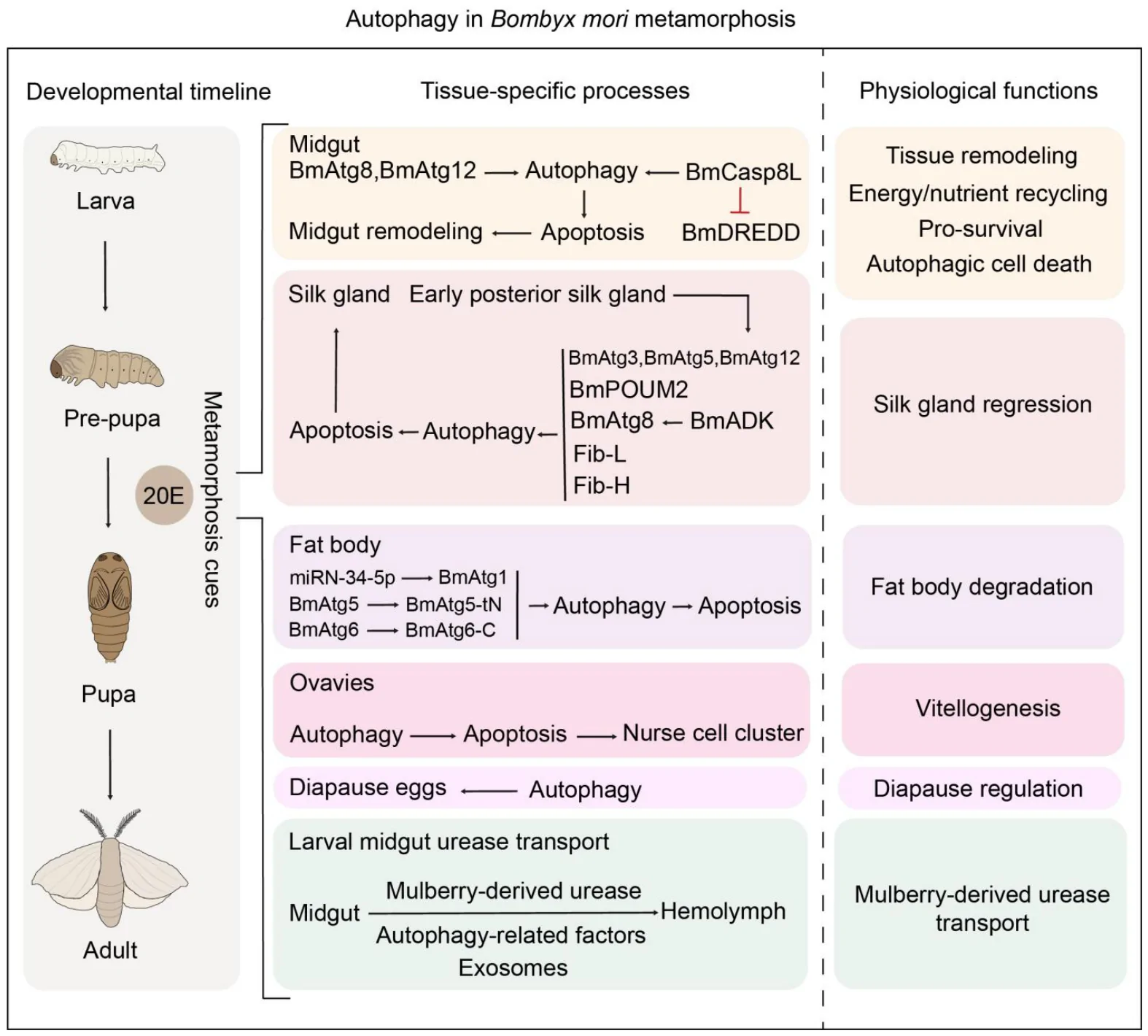
Autophagy plays pleiotropic roles in silkworm metamorphosis. BmCasp8L: acts as a positive regulator of autophagy by inhibiting BmDREDD-mediated cleavage of BmAtg6, thereby promoting autophagic flux; BmAtg5 and BmAtg6: their cleavage into BmAtg5-tN and BmAtg6-C mediates the transition from autophagy to apoptosis in the midgut during metamorphosis; BmAtg3, BmAtg5, and BmAtg12: essential for autophagy-mediated cell death in the posterior silk gland; BmADK: positively regulates autophagy in the silk gland, and its knockdown downregulates BmAtg8 expression; BmGDAP2: modulates developmental progression, likely via autophagy-related pathways, and its knockout prolongs the growth cycle; Fib-L (fibroin light chain): its mutation concurrently activates autophagy, apoptosis, and the heat shock response, leading to premature silk gland degradation; FibH (fibroin heavy chain): its truncated form in naked pupa (Nd) mutants disrupts cellular homeostasis and upregulates autophagy-related genes; POUM2: a transcription factor that regulates autophagy-related gene expression, influencing anterior silk gland remodeling and cell fate determination; miR-34-5p: a microRNA that participates in 20E-induced autophagy by targeting BmAtg1, linking miRNA regulation to 20E signaling and fat-body remodeling. Black arrows indicate promotion, red lines indicate inhibition, and different colors represent different tissues and organs.

**Figure 3:**
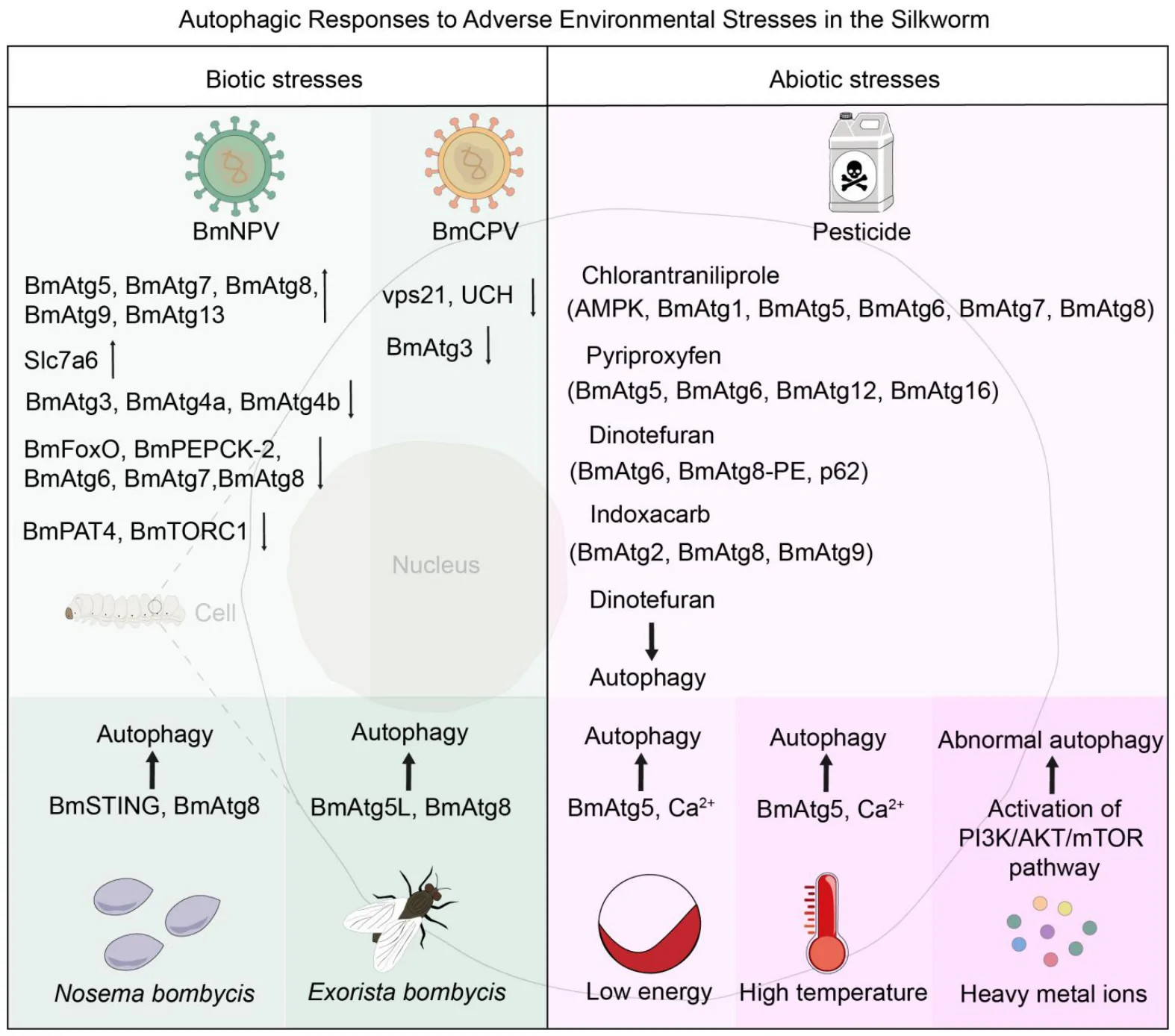
Schematic overview of autophagy regulation in silkworms in response to diverse environmental stressors. Autophagy in silkworm cells is triggered by a wide range of stimuli, including viral pathogens (e.g., BmNPV and BmCPV), chemical pollutants (e.g., pesticides such as chlorantraniliprole, pyriproxyfen, dinotefuran, and indoxacarb, as well as heavy metals), microbial parasites (*Nosema bombycis* and the parasitoid fly *Exorista bombycis*), and physicochemical stresses (e.g., starvation, high temperature, and nutritional imbalance). Thin arrows indicate the upregulation or downregulation of the relevant proteins; thick arrows indicate the activation of autophagy; different colors represent different types of stress.

**Table 1:** List of core autophagy-related genes characterized in silkworm.

Name	SilkDB NO.	Function	Reference
*BmAtg1*	BGIBMGA011986-TA	Ser/Thr kinase, required for autophagy	[34,35]
*BmAtg3*	BGIBMGA003767-TA	E2-like enzyme conjugates BmAtg8 to phosphatidylethanolamine (PE)	[38]
*BmAtg4*	BGIBMGA004926-TA	Cysteine protease, cleaves C-terminal extension or PE from BmAtg8	[44]
*BmAtg5*	BGIBMGA007878-TA	Conjugates to BmAtg12 through internal lysine	[39]
*BmAtg6*	BGIBMGA000092-TA	Component of class III PI3K complexes	[39]
*BmAtg7*	BGIBMGA001467-TA	E1-like enzyme activates BmAtg8 and BmAtg12	[40]
*BmAtg8*	BGIVMGA011783-PA	Conjugates with BmAtg7, BmAtg3	[42]
*BmAtg9*	BGIBMGA012307-PA	Transmembrane protein, formation of CVT and autophagic vesicles	[43]
*BmAtg12*	BGIBMGA003954-TA	Conjugates to BmAtg5 by E1 enzyme BmAtg7	[43]
*BmAtg13*	XP_004924339.1	Regulatory subunit of the BmAtg1 kinase complex	[46]
*BmAtg16*	BGIBMGA006504-TA	Protein interacts with BmAtg12–BmAtg5	[43]
*BmAtg18*	BGIBMGA007298-TA	Required for recycling of BmAtg9	[43]

## Data Availability

No new data were created or analyzed in this study. Data sharing is not applicable to this article.

## Abbreviations

20E	20-hydroxyecdysone
AMPK	AMP-activated protein kinase
Atg	Autophagy-related gene
BmCPV	*Bombyx mori* cytoplasmic nucleopolyhedrosis virus
BmNPV	*Bombyx mori* Nuclear Polyhedrosis Virus
CdTe-QDs	Cadmium telluride quantum dots
CMA	Chaperone-mediated autophagy
Fib-H	Fibroin heavy chain
Fib-L	Fibroin light chain
ISCs	Intestinal stem cells
mTOR	Mammalian Target of Rapamycin
NPC2	Niemann–Pick disease type C2
ROS	Reactive oxygen species
VPS	Vacuolar Protein Sorting
